# Storing fish?: a dog’s isotopic biography provides insight into Iron Age food preservation strategies in the Russian Arctic

**DOI:** 10.1007/s12520-020-01166-3

**Published:** 2020-08-03

**Authors:** Robert J. Losey, Eric Guiry, Tatiana Nomokonova, Andrei V. Gusev, Paul Szpak

**Affiliations:** 1 Department of Anthropology, University of Alberta, Edmonton, Canada; 2 School of Archaeology and Ancient History, University of Leicester, Mayor’s Walk, Leicester, UK; 3 Department of Anthropology, School of Archaeology and Ancient History, Trent University, Peterborough, Canada; 4 Department of Archaeology and Anthropology, University of Saskatchewan, Saskatoon, Canada; 5 Scientific Center of Arctic Studies, Iamal-Nenets Autonomous District, Salekhard, Russian Federation

**Keywords:** Arctic archeology, Stable isotope analysis, Dogs, Zooarchaeology, Paleodiet, Animal life history

## Abstract

Analysis of individual animal bodies can provide numerous useful insights in archeology, including how humans provisioned such animals, which in turn informs on a variety of other past behaviors such as human dietary patterns. In this study, we conducted stable carbon (*δ*^13^C) and nitrogen (*δ*^15^N) isotope analysis of collagen and keratin from four types of tissues from a dog burial at the Ust’-Polui site in the Iamal region of Arctic Russia. Ust’-Polui is an Iron Age site located on the Lower Ob River, a major northern fishery characterized by extreme seasonal shifts in fish presence. During a 6-month period stretching over the coldest months of the year, fish are nearly entirely absent in the Lower Ob River. Despite this, the stable isotope compositions of the dog’s bone and dentine collagen and hair and nail keratin all indicate a monotonous diet focusing on local fish. This pattern indicates the dog was provisioned year-round with fish. This was likely accomplished by mass harvesting of fish using nets or traps. Such fish were then processed and frozen for consumption during the non-fishing season. These findings suggest that people in the Ust’-Polui region also relied to some extent on fish throughout the year. Stored fish likely provided a dietary buffer for uneven returns from reindeer and bird hunting, both of which also are well-evidenced at the site.

**Electronic supplementary material:**

The online version of this article (10.1007/s12520-020-01166-3) contains supplementary material, which is available to authorized users.

## Introduction

**Fig. 1:**
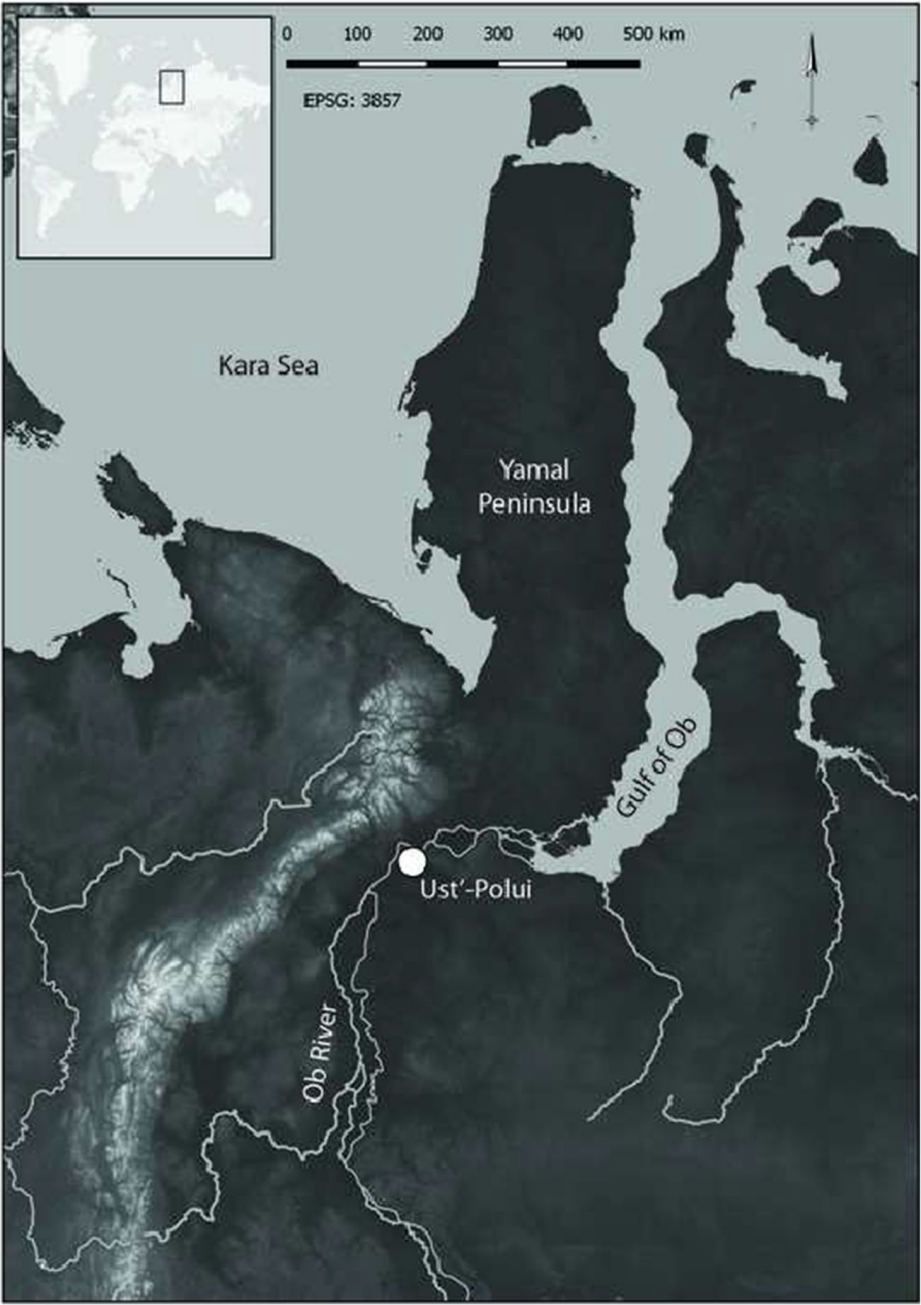
Map of the study area with the location of Ust’-Polui indicated

Archeological analyses of the remains of a single human can provide remarkable insights, particularly when atypical burial conditions allow for non-skeletal body tissues to preserve (c.f., Müller et al. 2003; Nystrom and Tilley 2019; Richards et al. 2007). Such insights can in turn be used to make inferences about broader patterns of behavior and the environment in which these persons participated. Individual animals offer the same potential (e.g., Tourigny et al. 2015), but most often, their remains are found disarticulated and fragmented, hindering such approaches. Intentionally made burials of domestic animals are an important exception to this pattern, as they offer analysts more complete bodies. Dogs are the most commonly buried animals (Morey 2006), and they also are the animals most often used as dietary proxies for humans in stable isotope studies (Guiry 2012, 2013; Guiry and Grimes 2013; McManus-Fry et al. 2018; Perri et al. 2019). In this study, we conducted stable carbon (*δ*^13^C) and nitrogen (*δ*^15^N) isotope analysis of collagen and keratin from four types of tissues from a dog buried at the Ust’-Polui site in Iamal (Fig. 1). This multi-tissue isotopic approach can help to develop a “biographical” perspective for reconstructing of this dog’s life and by extension, its interactions with humans (Losey et al. 2011). Isotopic compositions from the dog’s bone, tooth, hair, and nail tissues provide diachronic insights into the structure of its diet and how people provisioned this individual. Just as important, the dog’s isotopic compositions suggest the potential for year-round dietary reliance on fish by both people and dogs in the surrounding region.

Ust’-Polui is on the bank of the Ob River within the city of Salekhard, Russia, near the Arctic Circle (Fig. 1). Excavated since the 1930s (Adrianov 1936; Fedorova and Gusev 2008; Gusev and Fedorova 2012, 2017; Moshinskaia 1953; Moszyńska 1974), Ust’-Polui is one of the Siberian Arctic’s most spectacular sites. Modeling of multiple radiocarbon dates indicates it was occupied between ~ 260 BCE to 140 CE (Losey et al. 2017a). Ust’-Polui is interpreted to be a ritual site used by multiple regional communities, including for dog sacrifices and the deposition of implements, ornaments, and other materials as offerings. The site was surrounded on all but one side by a ditch and wood fence, with the remaining side terminating at the edge of the Ob River. Over 50,000 artifacts have been recovered from Ust’-Polui, and the intermittent permafrost deposits present there have produced numerous unique perishable items. The site also has what may be the earliest evidence for iron smelting anywhere in the Arctic (Vodyasov et al. 2017), as well as some of the oldest evidence for the use of domestic transport reindeer (Losey et al. 2020).

Ust’-Polui has also produced around 48,000 faunal remains (Bachura et al. 2017), which, along with the evidence for smelting, indicate that people resided at the site for some duration. Notably, no house remains were identified at the site, which in this general period are typically built within pits. Two human burials were also present, along with scattered human skeletal elements (Losey et al. 2017a). Most relevant here, the remains of at least 128 dogs have been recovered from Ust’-Polui, including several burials (Losey et al. 2018). These dogs likely participated in hunting and sled pulling, the latter evidenced by numerous sledding-related artifacts at the site. Many dogs also were butchered and eaten at the site, likely during ritual practices and feasting.

Iamal is one of the most extensively studied regions in the Russian Arctic, but subsistence and dietary patterns remain poorly understood. Written records from the last few centuries indicate that wild reindeer were a major dietary staple, with heavy dietary reliance on domestic reindeer emerging only in mid- to late eighteenth century CE (Krupnik 1993; Stépanoff 2017). The use of waterfowl and ptarmigan also is widely reported, the former being present during the warmer parts of the year, the latter available year-round. Fishing is widely described in ethnographic and historical sources, particularly on the Ob River, where traps, weirs, and nets were used for mass harvesting (Vizgalov et al. 2013: 130–159). The Ob fishery, however, was seasonally restricted due to a phenomenon locally known as *zamor*. During the *zamor* period, the river, most of its tributaries, and other bodies of water within the Ob flood plain experience a dramatic reduction of oxygen related to ice formation, influxes of organic matter, and reduced winter input of freshwater (Pavlov and Mocheka 2006; Popov 2017). These conditions persist for around 6 months, beginning with the buildup of ice around the end of October. To escape these conditions, nearly all fish in the Lower Ob migrate north into the central Gulf of Ob. It is only with the breakup of ice, typically in June, that fish begin to move back into the river, with the highest density occurring in the Ust’-Polui area in July and August (Pavlov and Mocheka 2006:57–60; Popov 2017). Small pockets of fish can be found in the *zamor* period only at the mouths of streams draining from the Ural Mountains to the west or at rare locations where springs offer well-oxygenated water (Vizgalov et al. 2013:220).

At Ust’-Polui, faunal remains suggest diets were relatively taxonomically diverse, at least when the site was utilized (Bachura et al. 2017). Sieves (4 mm mesh) were employed only during the 2009 excavations, and in these samples, fish remains account for half or more of the recovered specimens, followed in abundance by remains of mammals and birds. The dominant fish in the assemblage are burbot (*Lota lota*), with nelma (*Stenodus nelma*) and whitefish (*Coregonus* spp.) also being relatively abundant. These fish are common in the Ob River in the general site area, and nelma and whitefish historically constitute the core of this region’s modern commercial fishery (Vizgalov et al. 2013). Reindeer (*Rangifer tarandus*) and dogs (*Canis lupus familiaris*) constitute just over 84% of the mammal specimens in the total analyzed site sample, with hare (*Lepus timidus*) and beaver (*Castor fiber*) being the only other mammals present in more than trace quantities. Bird remains are very diverse but predominantly consist of waterfowl and ptarmigan (*Lagopus* spp.).

**Fig. 2:**
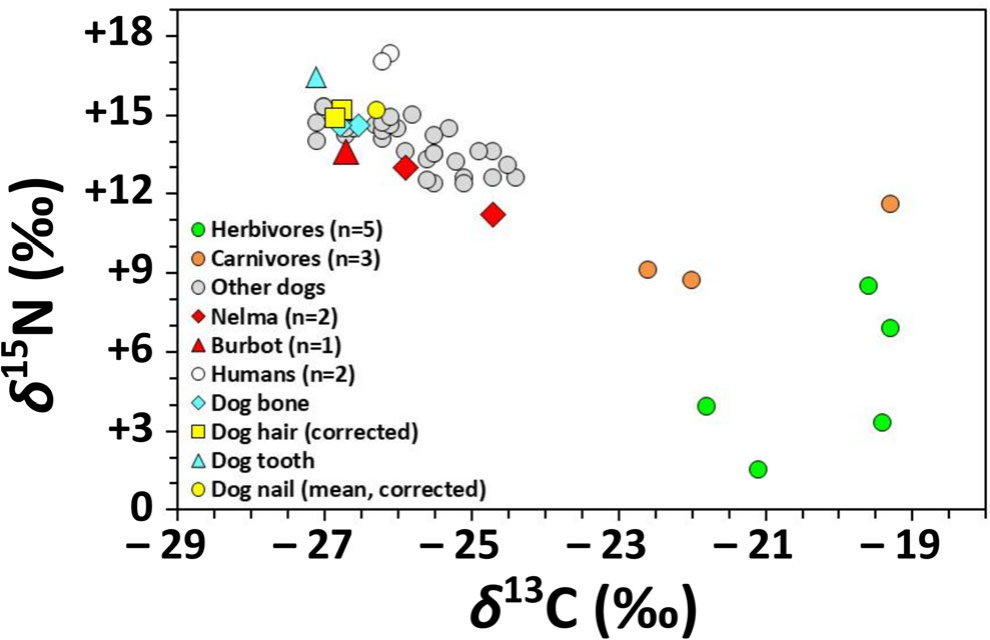
Stable carbon and nitrogen isotope values from humans and fauna from Ust’-Polui. The buried dog’s values generated in this study are shown in blue and yellow. Comparative data from Losey et al. (2017a)

Stable carbon and nitrogen isotope analyses of dog and human bone collagen from Ust’-Polui suggest both had long-term diets rich in aquatic foods, most likely freshwater fish (Losey et al. 2017a). The mean *δ*^13^C value for 32 adult dogs at the site, obtained on scapula or parietal samples, is − 25.8 ± 0.8‰ and the mean *δ*^15^N value is + 13.9 ± 0.9‰ (Fig. 2). Long bone diaphysis samples from two adult humans at the site had similar isotopic compositions, one with a *δ*^13^C value of − 26.1‰ and a *δ*^15^N value of + 17.3‰, and the other with a *δ*^13^C value of − 26.2‰ and *δ*^15^N of + 17.0‰. These values represent the diet for at least a 10-year period (Hedges and Reynard 2007). The only fauna analyzed from Ust’-Polui with similarly low *δ*^13^C and high *δ*^15^N values were nelma (*n* = 2) and burbot (*n* = 1), which together had a mean *δ*^13^C value of − 25.8 ± 1.0‰ and mean *δ*^15^N value of + 12.6 ± 1.3‰. Note that additional fish samples were subjected to stable isotope analyses but failed to pass quality standards. Regardless, because freshwater fish are the only local resource with low *δ*^13^C and high *δ*^15^N values, these data indicate the importance of local fish in both human and dog diets, but they provide no clear indication of how or if the structure of their diets varied seasonally.

To better assess seasonal variation in diets in the Ust’-Polui region, we analyzed the stable isotope compositions of a nearly complete dog skeleton found within partially frozen sediments. These burial conditions allowed for the recovery of some of the dog’s hair and nails. Incremental sampling of one tooth and one nail, along with bulk sampling of its hair and bone (a rib and distal phalange), provides glimpses of its diet over various periods of the animal’s life.

## Materials and methods

### Context and basic osteology

**Fig. 3:**
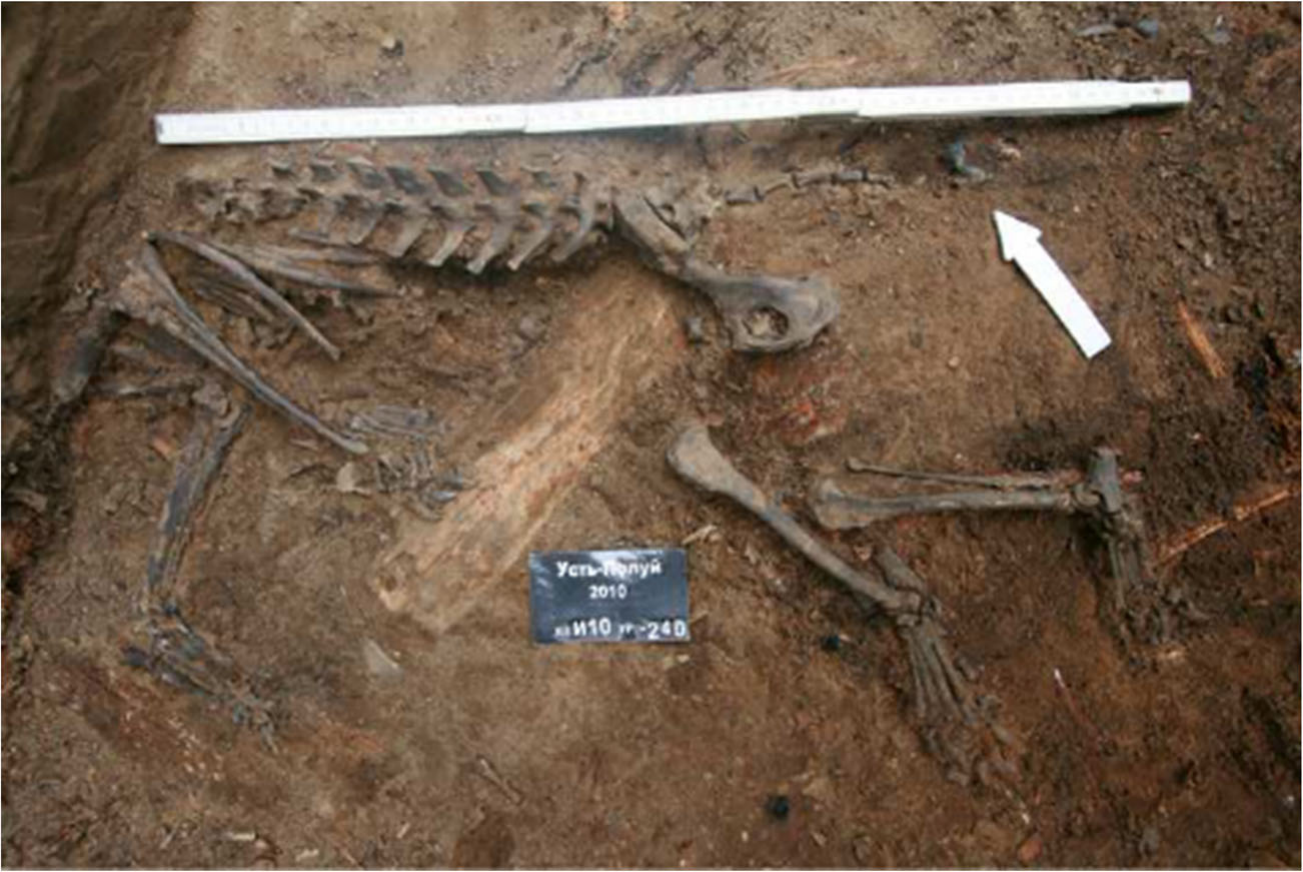
The analyzed dog burial at Ust’-Polui. Note that the dog’s head extended into an adjacent unit and was not yet excavated when photographed. Photo by A. Gusev

**Fig. 4:**
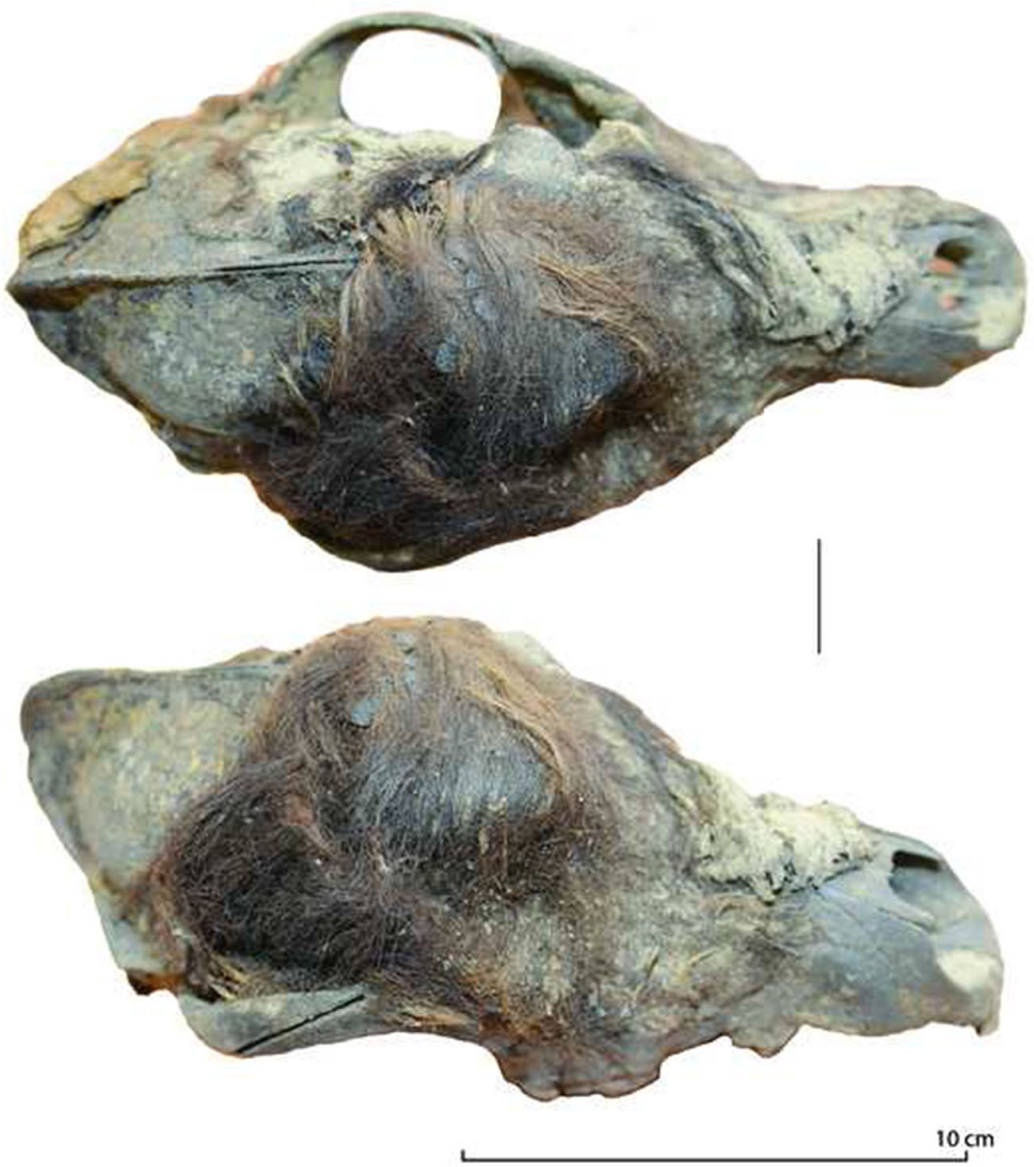
The cranium of the analyzed dog from Ust’-Polui showing the mat of hair found on upper right side. Photo by T. Nomokonova

The dog’s remains were found in the ditch area of the site (unit И10б level 240) in 2010 (Fig. 3). It was buried laying on its right side with its head oriented to the north-northwest. The skeleton was largely articulated, but some disturbance was noted, possibly due to post-depositional shifts in the sediment. Large clumps of hair and skin were found matted against the upper right side of the cranium and in the pelvic region (Fig. 4). Nails were found with nearly all of the digits (Fig. 3). No season of death information is available for the dog, but its burial at the site would have been most feasible during the warm season when snow was absent and the underlying soil unfrozen. After excavation, the specimen was displayed at the Iamal-Nenets Region Museum Complex of I.S. Shemanovsky in Salekhard, rendering it inaccessible to us in previous studies.

The dog skeleton was visually examined for tooth loss, pathological lesions, and tooth wear, and its skeletal dimensions were measured with a sliding caliper following Von den Driesch (1976). Age estimation was based on skeletal element fusion and tooth eruption (Evans and de Lahunta 2012; von Pfeil and DeCamp 2009). Body mass was estimated from cranium length (Losey et al. 2017b) and withers height from humerus length (Harcourt 1974).

### Isotopic analyses

#### Bone collagen

Bone collagen was extracted from a rib and distal phalange as follows. Bone samples were demineralized in 0.5 M HCl. Samples were then neutralized in Type 1 water and base-soluble contaminants (e.g., humic acids) were removed by soaking in 0.1 M NaOH in an ultrasonic bath (solution refreshed every 15 min until solution remained clear). Samples were then neutralized again in Type 1 water and refluxed in 0.01 M HCl solution at 65 °C for 36 h. Samples were centrifuged, and the solubilized fraction pipetted to a fresh tube, which was then frozen and lyophilized.

Bones remodel at different rates due to a wide range of factors such as bone density, biological age, and activity levels (Hedges et al. 2007; Stenhouse and Baxter 1979). Bone collagen turnover rates may vary between ribs and distal phalanges (Clark et al. 2017). For this reason, we anticipate that isotopic compositions from bone collagen will reflect an average of multiple years of dietary intake, although perhaps weighted towards slightly different time frames among skeletal elements. More variable isotopic compositions among elements may therefore be associated with high levels of temporal variation in diet.

#### Tooth collagen

In contrast to bone, a wider range of methods, with large differences in temporal resolutions, have been developed for tooth serial sampling and collagen extraction (for review see Guiry et al. 2016). The serial sampling method selected reflects a compromise between temporal resolution and collagen quality control. The highest resolution techniques typically use collagen extracted from minute powdered dentine samples drilled from rasters situated perpendicular to a tooth’s growth axis (e.g., Rossman et al. 2015). However, these methods do not provide an opportunity for removal of base-soluble contaminants, and, it should therefore be avoided when sampling teeth that may have elevated potential for burial environment contamination (Guiry et al. 2018). Based on previous analyses suggesting that there may be elevated potential for humic acid contamination at this site (Losey et al. 2017a), we selected an alternative method allowing for the application of NaOH step to remove base-soluble contaminants.

The tooth, a right third maxillary incisor, was halved along its sagittal plane using a dental cut wheel and one half was demineralized in 0.5 M HCl. After demineralization, the tooth became pliable and elastic, allowing for easier manipulation and sub-sampling using a scalpel blade. The demineralized tooth half was then neutralized in Type 1 water and flattened by scoring with two incisions made parallel with its growth axis (from root apex to crown tip). The flattened tooth was then divided into sub-samples using a scalpel to serially excise slivers of dentine (cutting from the internal to external surface) perpendicular to the tooth’s growth axis at an oblique angle approximating the growth angle of dentine layers during tooth development. Due to the complex architecture of this tooth (with curvatures occurring on *x*, *y*, and *z* axes), it was not practical to incorporate information for tooth histology when selecting the angle (i.e., the relative obliqueness) at which samples were taken. While the serial sampling approach used here (i.e., cutting at generalized oblique angles) will be inherently less precise than methods that incorporate bespoke cutting patterns that are customized to a sample based on histological observations, it has the benefit of being less resource intensive. It also should still help to obviate some of the issues associated with sampling strategies that do not take the oblique angle of dentine deposition layers into account. Collagen extractions from dentin sub-samples then proceeded in the same manner as described above for demineralized bone collagen samples.

While radiographic analyses have explored a wide range of dog tooth formation processes (e.g., Cahill and Marks Jr 1982; Morgan and Miyabayashi 1991; Williams and Evans 1978), there has been relatively little analysis documenting the formation periods for adult dog dentition (for review, see Evans and de Lahunta 2012: 32–22, 285–289). From the limited data available in published literature (Arnall 1960), we anticipate that the isotopic time series from this dog tooth will span from 2 to 3 weeks after birth to about 6 months of age.

#### Hair keratin

Analysis of bulk hair keratin, rather than serial samples, was chosen because the relatively short length (ca. < 20 mm) of the individual dog hairs rendered them unsuitable for intra-hair sequential sampling. Hair sample clumps from pelvic and cranial areas were cleaned with successive rinses (5 min each) in Type 1 water, 2:1 chloroform:methanol (Folch et al. 1957), and Type 1 water in an ultrasonic bath to remove adhering lipids and other contaminants. Hair samples were then air dried.

A wide range of factors can affect the rate at which dog hairs grow, including season, region of body, and nutrition (Gunaatnam and Wilkinson 1983). For this reason, we conservatively estimate that hair samples will have isotopic compositions reflecting a period of between 2 to 3 weeks and 3 months. Also, based on some limited experimental evidence suggesting that dog hair growth rates differ between forehead and flank regions (Gunaatnam and Wilkinson 1983), we anticipate that cranial hair samples may represent a slightly shorter period than the pelvic hair samples.

#### Nail keratin

Our approach for serial sampling of nail was conceptually similar to that for tooth dentin and followed Kielland (2001), modified as follows. A thin strip (ca. 1-mm wide) parallel with the nail’s growth axis was removed from the superior surface (i.e., extending from proximal to distal end) using a dental cut wheel. The interior and exterior surfaces of the strip of nail were then lightly abraded with a dental burr in order to remove any adhering non-keratinous contaminants. A scalpel was used to divide the strip of nail into sub-samples by successively shaving off keratin slivers (cutting from the internal to external surface) at an oblique angle approximating the angle of growth of keratin layers during nail development.

A wide variety of factors influence the growth rate of dog nails, including age and nutrition, but based on the length of the nail (ca. 20 mm) and the observed growth rate in domestic dogs (ca. 0.8–2.1 mm/week, Orentreich et al. 1979), we anticipate that the isotopic time series obtained from this nail will reflect the last 1.5 to 6 months of life.

#### Isotopic analyses

Stable carbon and nitrogen elemental and isotopic compositions of 0.5 mg sub-samples of keratin and collagen were measured on an Elemental Analyzer 300 (Eurovector, Pavia, Italy) coupled via continuous flow to a Horizon Isotope Ratio Mass Spectrometer (Nu Instruments, Wrexham, UK) at the Water Quality Center at Trent University. Hair keratin samples were measured in triplicate (anticipating greater potential for isotopic heterogeneity) and bone collagen samples in duplicate. It was not possible to perform replicate analyses of nail sub-samples. *δ*^13^C and *δ*^15^N were calibrated using a two-point calibration curve anchored by USGS40 and USGS41a relative to VPDB and AIR, respectively (Electronic Supplementary Materials [ESM]1, Table S1; Qi et al. 2003; Qi et al. 2016). Analytical accuracy was monitored using three internal check standards (see ESM, Table S1). Standard deviations and numbers for sample replicates, calibration standards, and check standards are provided in ESM Tables S2, S3, and S4, respectively. Following Szpak et al. (2017), for *δ*^13^C and *δ*^15^N, respectively, we calculated random errors (*uR*_*(w)*_) to be ± 0.07‰ and ± 0.15‰; systematic errors (*u*_*(bias)*_) to be ± 0.09‰ and ± 0.17‰; and standard uncertainty to be ± 0.11‰ and ± 0.23‰. Collagen quality was assessed using the following well-established criteria: C:N_Atomic_ (2.9–3.6), %C (> 13.0%), and %N (> 4.5%) (Ambrose 1990; DeNiro 1985). Although quality control indicators for archeological keratin have not been established for hair and nail samples, we use the C:N_Atomic_ range (3.0–3.8) observed for modern samples to assess keratin quality (Szpak and Valenzuela 2020).

## Results

The Ust’-Polui dog had a fully fused post-cranial skeleton indicating that it was over 1 year of age. The teeth exhibited slight wear, and the left mandibular fourth premolar was lost premortem, with the alveolus filled with new bone, also suggesting the animal was an adult. The estimated body mass is 18.5 ± 2.6 kg, meaning the dog was slightly above the mean estimated adult body mass of dogs at the site, which is 16.8 ± 3.4 kg (Losey et al. 2018). Its estimated withers height is 50.2 cm, nearly identical to the mean for the site (Losey et al. 2018). No *os penis* is visible in the excavation photos, nor is one present with the curated remains, suggesting the dog was female. The dog suffered trauma to its right lower back/hip area, likely several weeks or months before death, fracturing the right transverse process of its seventh lumbar vertebra, and damaging the right cranial wing of the sacrum. Both exhibit new bone growth, indicating that healing was underway at death.

All stable isotope samples had %C, %N, and C:N_Atomic_ falling within quality control criteria for acceptability (Table 1). Isotopic compositions differed little between rib and phalanx bone collagen samples (for *δ*^13^C, mean = − 26.7‰, difference = 0.3‰; *δ*^15^N, mean = + 14.6‰, difference > 0.1‰) (Fig. 2). These bone collagen isotopic compositions provide a baseline for longer-term dietary intake over the latter portion of this dog’s life.

**Table 1:** Stable carbon and nitrogen isotope values for the Ust’-Polui dog burial

TEAL no.	Material	*δ*^13^C	*δ*^15^N	%C	%N	C:N	Segment	% Col. Yld.
9808	Rib #3	− 26.5	+ 14.6	41.3	15.6	3.10	NA	11.9
9809	Phalange	− 26.8	+ 14.6	40.6	15.2	3.12	NA	13.6
9810	Dog maxillary 3rd incisor	− 27.1	+ 16.5	43.8	16.0	3.20	NA	NA
9810.01	Dog maxillary 3rd incisor	− 26.9	+ 15.4	29.4	10.7	3.21	1	NA
9810.02	Dog maxillary 3rd incisor	− 26.3	+ 15.7	36.5	13.3	3.20	2	NA
9810.03	Dog maxillary 3rd incisor	− 25.7	+ 15.2	31.5	11.5	3.21	3	NA
9810.04	Dog maxillary 3rd incisor	− 26.5	+ 15.8	40.9	14.9	3.19	4	NA
9810.05	Dog maxillary 3rd incisor	− 26.6	+ 15.9	40.1	14.6	3.20	5	NA
9810.06	Dog maxillary 3rd incisor	− 26.7	+ 16.1	42.7	15.6	3.19	6	NA
9810.07	Dog maxillary 3rd incisor	− 26.6	+ 16.1	41.2	15.1	3.18	7	NA
9810.08	Dog maxillary 3rd incisor	− 27.0	+ 16.2	42.6	15.6	3.19	8	NA
9810.09	Dog maxillary 3rd incisor	− 26.4	+ 15.9	36.8	12.8	3.36	9	NA
9810.10	Dog maxillary 3rd incisor	− 27.2	+ 16.4	42.0	15.5	3.17	10	NA
9810.11	Dog maxillary 3rd incisor	− 27.6	+ 16.7	41.3	15.1	3.18	11	NA
9810.12	Dog maxillary 3rd incisor	− 27.7	+ 16.7	37.3	13.7	3.18	12	NA
9810.13	Dog maxillary 3rd incisor	− 27.7	+ 16.6	42.4	15.6	3.16	13	NA
9810.14	Dog maxillary 3rd incisor	− 27.7	+ 16.6	38.8	14.2	3.19	14	NA
9810.15	Dog maxillary 3rd incisor	− 27.3	+ 16.8	38.5	14.0	3.20	15	NA
9811	Nail	− 28.2	+ 14.6	38.3	12.9	3.46	1	NA
9811	Nail	− 28.3	+ 14.8	27.1	9.1	3.47	2	NA
9811	Nail	− 28.5	+ 14.6	25.5	8.7	3.41	3	NA
9811	Nail	− 28.2	+ 14.5	32.7	11.1	3.45	4	NA
9811	Nail	− 28.1	+ 14.3	30.7	10.5	3.42	5	NA
9811	Nail	− 28.0	+ 14.2	40.5	13.5	3.49	6	NA
9811	Nail	− 27.9	+ 14.2	32.7	11.1	3.44	7	NA
9811	Nail	− 27.7	+ 13.8	34.9	11.8	3.45	8	NA
9811	Nail	− 27.6	+ 13.8	38.1	12.9	3.45	9	NA
9811	Nail	− 27.6	+ 13.9	34.4	11.5	3.47	10	NA
9811	Nail	− 27.7	+ 14.9	37.0	12.5	3.46	11	NA
9811	Nail	− 27.6	+ 13.9	37.5	12.5	3.51	12	NA
9811	Nail	− 27.6	+ 14.0	38.8	13.1	3.45	13	NA
9811	Nail	− 27.6	+ 13.8	30.1	10.1	3.48	14	NA
9811	Nail	− 27.6	+ 14.1	26.2	8.9	3.43	15	NA
9811	Nail	− 27.5	+ 14.7	52.6	17.4	3.53	16	NA
9811	Nail	− 27.1	+ 13.7	45.5	15.0	3.53	17	NA
9811	Nail	− 26.6	+ 14.2	24.4	8.0	3.57	18	NA
9811	Nail	− 27.9	+ 14.0	35.6	11.8	3.51	19	NA
9811	Nail	− 27.8	+ 13.9	55.8	18.1	3.59	20	NA
9811	Nail	− 26.6	+ 13.8	41.4	13.7	3.53	21	NA
9811	Nail	− 27.6	+ 14.0	22.5	7.3	3.57	22	NA
9812	Pelvic hair	− 28.2	+ 14.3	34.2	11.5	3.46	NA	NA
9813	Cranial hair	− 28.3	+ 14.0	31.7	10.7	3.45	NA	NA

*NA* not applicable

In the context of faunal baseline stable isotope compositions from this site (Losey et al. 2017a) (Fig. 2), bone collagen *δ*^13^C and *δ*^15^N for this dog demonstrate that it had maintained a highly specialized diet, and one quite similar to that of the other analyzed dogs. The dog’s isotopic compositions are far outside of the baseline range for local terrestrial herbivores (*n* = 5, mean *δ*^13^C = − 20.2 ± 1.1‰, mean *δ*^15^N = + 4.8 ± 2.8‰) and carnivores (*n* = 3, mean *δ*^13^C = − 21.3 ± 1.8‰, mean *δ*^15^N = + 9.8 ± 1.5‰) and, instead, fall squarely within the range that would be expected for animals with a diet primarily focused on local fish (*n* = 3, mean *δ*^13^C = − 25.8 ± 1.0‰, mean *δ*^15^N = + 12.6 ± 1.3‰). Although the local fish baseline is small, the similarity in data from two species with divergent habitat preferences, burbot (a deeper-water, bottom-associated fish) and nelma (a pelagic and highly migratory fish, but in this case potomodromous; see Guiry et al. 2020a), suggests that fish from a wide variety of ecosystems in the region likely also have highly ^13^C-depleted and ^15^N-enriched isotopic compositions. Such compositions appear to reflect distinctive characteristics of the local aquatic carbon and nitrogen sources and cycling (for similar a larger regional example see Guiry et al. 2020b).

**Fig. 5:**
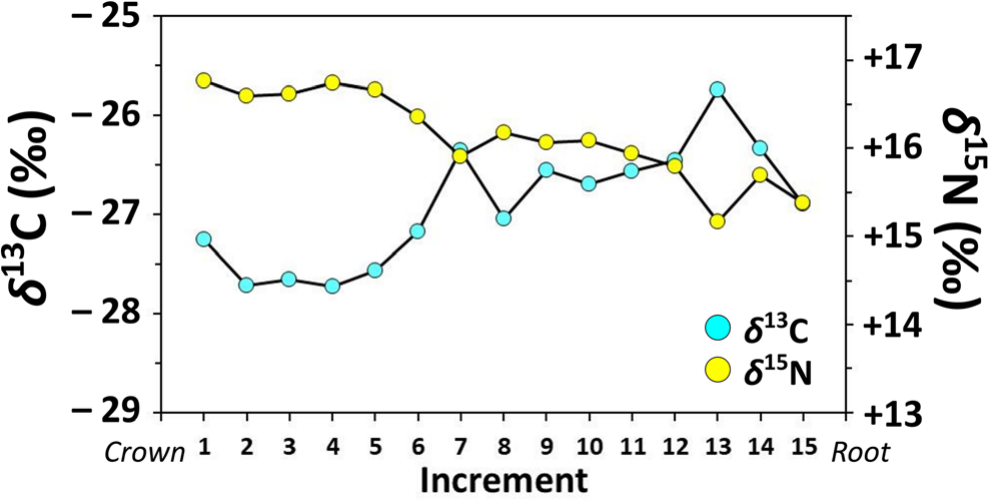
Stable carbon and nitrogen isotope values of dentine from a serially sampled upper 3rd incisor from the dog burial at Ust’-Polui

In contrast to bone, tooth dentine collagen isotopic compositions provide a record of diet during early life. Mean isotopic compositions for tooth dentine serial samples (*n* = 15; *δ*^13^C = − 26.9 ± 0.6‰; *δ*^15^N = + 16.8 ± 0.5) are similar to those of the bulk dentine samples (*δ*^15^N = + 16.5‰; *δ*^13^C = − 27.1‰) and, in comparison with bone collagen (with a similar *δ*^13^C, but much lower *δ*^15^N), suggest that this dog was nursing during part of the tooth’s formation (Fig. 2). Isotopic compositions of serially sampled dentine collagen range 2.0‰ (from − 25.7 to − 27.7‰) and 1.6‰ (from + 15.2 to + 16.8‰) for *δ*^13^C and *δ*^15^N, respectively (Fig. 5). Dentine collagen *δ*^15^N was highest at the crown but began to decrease after the fifth sample increment, suggesting that this dog likely began the weaning process sometime during its second month. However, we must emphasize that without more accurate tooth formation time estimates for dogs (see “Materials and methods”), this estimation of weaning age should be considered tentative. As with dentine collagen *δ*^15^N, *δ*^13^C of the first five sample increments showed little variation, consistent with a homogenous milk-based diet. Moving towards the root (later forming), *δ*^13^C of the remaining ten dentine samples was much more variable, with an overall upward trend suggesting that the pup was weaned onto somewhat less ^13^C-depleted foods similar to those of adult diet (i.e., as per bone collagen).

Keratin and bone collagen isotopic compositions systemically differed from one another. Following O'Connell et al. (2001), we correct keratin isotopic compositions by + 1.4‰ for *δ*^13^C (*δ*^13^C_Corrected_) and + 0.9‰ for *δ*^15^N (*δ*^15^N_Corrected_) to make them comparable with bone collagen. Isotopic compositions for hair taken from pelvic and cranial (forehead) regions showed little isotopic variation (for *δ*^13^C_Corrected_, mean = − 26.8‰, difference = 0.1‰; *δ*^15^N_Corrected_, mean = + 15.1‰, difference = 0.3‰) and provided a “snapshot” of diet in the last several weeks of life (Fig. 2). Mean bone collagen and corrected hair keratin isotopic compositions differ by only 0.1‰ and 0.5‰ for *δ*^13^C and *δ*^15^N, respectively, suggesting that diet in the final month of life was isotopically similar (i.e., fish-focused) to that of a large portion of the dog’s adult and, likely, later juvenile life.

**Fig. 6:**
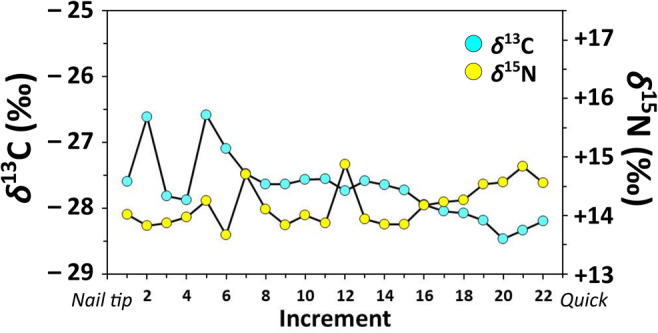
Stable carbon and nitrogen isotope values of nail keratin from the dog burial at Ust’-Polu

In contrast to hair samples, nail keratin isotopic compositions provided a record of the last several months of the dog’s life. Mean isotopic compositions for nail keratin serial samples (*n* = 22; *δ*^13^C_Corrected_ = − 26.3‰; *δ*^15^N_Corrected_ = + 15.2) are similar to those of hair keratin (*δ*^13^C_Corrected_ = − 26.8‰; *δ*^15^N_Corrected_ = + 15.1‰) (Fig. 2). Isotopic compositions of serially sampled nail keratin range 1.9‰ (from − 25.2 to − 27.1‰) and 1.2‰ (from + 14.6 to + 15.8‰) for *δ*^13^C_Corrected_ and *δ*^15^N_Corrected_, respectively (Fig. 6), suggesting that this dog’s diet was also relatively isotopically homogenous (i.e., still fish-focused) over the last several months of its life. Slight, although inconsistent trends appear to occur in these nail keratin isotopic compositions with a minor decrease and increase in *δ*^13^C and *δ*^15^N, respectively, which would be consistent with small adjustments in the proportion of diet made up by fish, the species of fish consumed, or spatiotemporal variation in local aquatic baselines (for review, see Guiry 2019).

## Discussion and conclusion

Taken together, the stable isotope results indicate that the dog derived the bulk of its dietary protein from fish, likely from the local area. By comparing results from four different tissues, which record diet during early and later life and at a variety of temporal scales, we can also see that the dog’s dietary focus on fish was somewhat monotonous, involving consumption of foods that were isotopically homogenous over much of its lifetime. The similarity between isotopic compositions measured from this dog’s tissues and bone collagen from 32 other dogs from the same site (Losey et al. 2017a) further suggests that this pattern of dietary monotony may have been widespread for dogs in the region. More broadly, the apparent lack of dietary variation, coupled with the intensive focus on fish, demonstrates that this dog was likely heavily dependent on humans for foods year-round, an isotopic pattern widely observed across the globe (Guiry 2012), particularly in northern regions (Guiry and Grimes 2013).

These results are intriguing given that the Ob River and other regional bodies of freshwater were largely devoid of fish for half of the year due to the *zamor* phenomenon described earlier. In this context, there are two possible explanations for the dog’s apparent year-round reliance on fish.

First, it is possible this dietary patterning was accomplished by moving to locations where fish remained abundant during the *zamor*. This seems possible for at least some local groups, which might be able to access the remaining fish populations by chipping through the ice on the rivers draining from the Ural Mountains to the west. Few such rivers are present locally, and suitable locations on them for winter fishing (deep sections) would have been limited. Following fish downstream as they migrated out of the Ob and its tributaries is another possibility, but this would require long-distance movement. By January, for example, the *zamor* pushes fish at least 200 km to the northeast into the Gulf of Ob, and by May, this extends northward at least another 100 km (Pavlov and Mocheka 2006:57–60; Popov 2017).

The second, and most parsimonious explanation, is that fish were taken locally in large numbers and stored for use as year-round dog food. The fishing season would end with the onset of cold weather in the fall and advent of the *zamor*. Fortuitously, these same conditions would have been highly conducive to fish preservation. The modern mean high temperature in Salekhard, where Ust’-Polui is located, remains below 0 °C from October through much of May (Hydrometcenter of Russia 2020), meaning that fish could be frozen for storage for the *zamor*—laborious smoking or salting was not required. Climate data suggest that summer temperatures in Iamal were somewhat cooler than present at the time Ust’-Polui was occupied (Briffa et al. 2013). Air drying and freezing of fish for long-term use of dog food is also documented for the Lower Ob region in the historic period (Perevalova 2004). Furthermore, fish mass harvesting technology was extensively utilized in the region historically and is evidenced long prior to the occupation of Ust’-Polui, with gill nets appearing by ~ 3000 BCE (Tupakhina and Tupakhin 2018). A few net sinkers have been recovered from Ust’-Polui itself (Gusev and Fedorova 2017), also indicating the likelihood of intensive fish harvesting. People inhabiting the Ust’-Polui area also potentially had diets rich in fish year-round. Recall that the stable isotope values for two humans buried at the site are similar to those of the dogs, albeit with somewhat higher *δ*^15^N values—their long-term diets included significant amounts of fish (Losey et al. 2017a). If fish could be frozen and stored for dogs well beyond the fishing season, they could be kept in this same way for human consumption. Such stored fish could have formed a staple in regional diets that could be relied upon in the face of inconsistent returns from other subsistence pursuits, such as reindeer or waterfowl hunting. These faunae too varied seasonally in abundance and quality, and stored fish could have helped to buffer such variations, as seen in other northern environments (Harris et al. 2020; McManus-Fry et al. 2018).

An additional fish preservation method to consider is fermentation. This process often involves the use of bacteria to introduce lactic acid into foods, with the acid acting to prevent the development of harmful bacteria (Campbell-Platt 1987). Fish fermentation has been widely practiced historically, and appeared in some regions by at least 9000 years ago (Boethius 2016). Among many northern Indigenous societies, this process was relatively simple and economical. It involved placing foods in lined pits in the ground, or placing foods in skin bags that were submerged in water; both procedures helped ensure the process of fermentation would occur under cool temperatures (Speth 2017). Fermentation has the advantages of requiring no fuel and little food processing (as needed in most of fish-smoking processes), and is known to have some nutritional benefits (Boethius 2016; Speth 2017; Doering 2019) as well as culturally significant flavor profiles (e.g., Yamin-Pasternak et al. 2014).

There are, however, a number of factors that suggest fish fermenting provides a less parsimonious explanation for fish preservation at Ust’-Polui. First, under the climatic conditions that would likely have prevailed around the time that fish were mass captured at Ust’-Polui, freezing could arguable represent a less laborious process for fish preservation than fermentation (which would require sustained temperatures above freezing). Second, ethnographically, basic methods of fish fermentation are seemingly most commonly practiced in the North where damp conditions inhibit smoke drying, or where wood fuel is scarce (Speth 2017). Ust’-Polui is in the forest-tundra where dwarf trees are abundant, and large quantities of wood drift northward into the region from the Ob’ River and its tributaries (Vizgalov et al. 2013). For this reason, in addition to low processing methods such as freezing, wood was surely readily available in the study area, should more laborious smoking and drying processes have been a preferred method for preservation. Third, fish fermentation is currently not practiced in Iamal, and we are unaware of any historical accounts of it in the region. We are also unaware of any archeological features in Iamal that have been interpreted as fermentation pits. Finally, while not ruled out by our analyses, invoking consumption of fermented fish, which would have lower *δ*^13^C and higher *δ*^15^N relative than their un-fermented counterparts (Doering 2019), is not required to explain the observed isotopic compositions of dogs at Ust’-Polui (i.e., they are easily accounted for by consumption of non-fermented foods within the context of our isotopic baseline from local fauna). We nonetheless cannot rule out the possibility that fermentation was used in conjunction with other methods for preserving fish at Ust’-Polui.

As long-term storage of frozen fish for winter consumption would obviate the need to undertake costly long-distance journeys to follow migrating fish, and would be easily achieved under local climatic conditions, we believe that this dog’s year-round fish-focused diet most likely reflects the importance of stored frozen fish in the Ust’-Polui area during the Iron Age. Further diachronic analysis of multiple tissues of additional dogs in this and other regions will help to establish how widespread this practice may have been and to better document local and seasonal variation in dog diet and provisioning practices. Incremental sampling of human dentition for stable isotope analysis can generate dietary information over several years, potentially providing additional lines of evidence for assessment of seasonal dietary variation. Archeological surveys of locations considered less affected by *zamor* fish displacement could also be beneficial, particularly along the major rivers stemming from the Urals and the west-central coastline of the Gulf of Ob. Finally, additional sampling of fish from Ust’-Polui should aid in determining which species were most critical to human and dog diets in the region.

## Electronic supplementary material

ESM 1 (DOCX 38 kb)
