# Supplementary material for: Storing fish?: a dog’s isotopic biography provides insight into Iron Age food preservation strategies in the Russian Arctic
Source: Archaeol Anthropol Sci. 2020 Aug 3;12(8):200. doi: 10.1007/s12520-020-01166-3 (PMC7410107; doi:10.1007/s12520-020-01166-3)
Supplement: Supplementary file 1 — (DOCX 38 kb) [file 12520_2020_1166_MOESM1_ESM.docx]

**Electronic Supplementary Information**

**Table S1**. Accepted (calibration) and observed long-term (check) isotopic compositions and standard deviations (1σ) for standards used in this study.

| **Name** | **Material** | **Number** | ***δ*^13^C (‰, VPDB)** | ***δ*^15^N (‰, AIR)** | **Standard Type** |
| --- | --- | --- | --- | --- | --- |
| USGS40 | Glutamic acid | NA | −26.39 | −4.52 | Calibration standard |
| USGS41 | Glutamic acid | NA | +37.63 | +47.57 | Calibration standard |
| MET | Methionine | 1046 | −28.62±0.11 | −5.03±0.15 | Check standard |
| SRM-15 | Deer bone collagen | 104 | −26.88±0.05 | +6.90±0.08 | Check standard |
| SRM-16 | Seal bone collagen | 132 | −14.81±0.10 | +16.91±0.08 | Check standard |

**Table S2**. Standard deviations for calibration standards for all analytical sessions.

| **Analytical Session** | **Standard** | **Number** | ***δ*^13^C (1*σ*)** | ***δ*^15^N (1*σ*)** |
| --- | --- | --- | --- | --- |
| CN19-26 | USGS40 | 9 | 0.03 | 0.08 |
| CN19-29 | USGS40 | 7 | 0.03 | 0.06 |
| CN20-04 | USGS40 | 9 | 0.02 | 0.10 |
| CN20-05 | USGS40 | 10 | 0.03 | 0.12 |
| CN19-26 | USGS41a | 9 | 0.06 | 0.28 |
| CN19-29 | USGS41 | 8 | 0.05 | 0.14 |
| CN20-04 | USGS41a | 9 | 0.06 | 0.07 |
| CN20-05 | USGS41a | 9 | 0.04 | 0.26 |

**Table S3**. Means and standard deviations for check standards for all analytical sessions.

| **Standard** | **Analytical Session** | **Number** | ***δ*^13^C (1*σ*)** | | | ***δ*^15^N (1*σ*)** | | |
| --- | --- | --- | --- | --- | --- | --- | --- | --- |
| MET | CN19-26 | 7 | -28.60 | ± | 0.02 | -5.27 | ± | 0.14 |
| MET | CN19-29 | 5 | -28.59 | ± | 0.04 | -5.09 | ± | 0.12 |
| MET | CN20-04 | 7 | -28.61 | ± | 0.03 | -5.13 | ± | 0.13 |
| MET | CN20-05 | 7 | -28.60 | ± | 0.03 | -5.01 | ± | 0.05 |
| SRM-15 | CN19-26 | 6 | -26.91 | ± | 0.03 | 6.80 | ± | 0.13 |
| SRM-15 | CN19-29 | 6 | -26.87 | ± | 0.04 | 6.83 | ± | 0.11 |
| SRM-15 | CN20-04 | 6 | -26.89 | ± | 0.04 | 6.82 | ± | 0.18 |
| SRM-15 | CN20-05 | 7 | -26.89 | ± | 0.03 | 6.90 | ± | 0.05 |
| SRM-16 | CN19-26 | 4 | -14.85 | ± | 0.04 | 17.09 | ± | 0.06 |
| SRM-16 | CN19-29 | 5 | -14.83 | ± | 0.02 | 16.88 | ± | 0.09 |
| SRM-16 | CN20-04 | 5 | -14.79 | ± | 0.02 |  |  |  |
| SRM-16 | CN20-05 | 5 | -14.82 | ± | 0.04 | 17.00 | ± | 0.12 |

**Table S4**. Standard deviations for sample replicated from all analytical sessions.

| **Sample** | ***δ*^13^C (A)** | ***δ*^13^C (B)** | ***δ*^13^C (C)** | ***δ*^13^C (1*σ*)** | | | ***δ*^15^N (A)** | ***δ*^15^N (B)** | ***δ*^15^N (C)** | ***δ*^15^N (1*σ*)** | | |
| --- | --- | --- | --- | --- | --- | --- | --- | --- | --- | --- | --- | --- |
| TEAL 9808 | -26.52 | -26.55 |  | -26.53 | ± | 0.02 | 14.54 | 14.69 |  | 14.62 | ± | 0.11 |
| TEAL 9809 | -26.79 | -26.78 |  | -26.79 | ± | 0.01 | 14.61 | 14.63 |  | 14.62 | ± | 0.01 |
| TEAL 9810 | -27.13 | -27.11 |  | -27.12 | ± | 0.01 | 16.42 | 16.49 |  | 16.46 | ± | 0.05 |
| TEAL 9812 | -28.09 | -28.33 | -28.07 | -28.17 | ± | 0.14 | 14.30 | 14.41 | 14.24 | 14.32 | ± | 0.09 |
| TEAL 9813 | -28.34 | -28.21 | -28.25 | -28.26 | ± | 0.07 | 14.05 | 14.05 | 13.91 | 14.00 | ± | 0.08 |
